# Supplementation with Hydroxytyrosol and Punicalagin Improves Early Atherosclerosis Markers Involved in the Asymptomatic Phase of Atherosclerosis in the Adult Population: A Randomized, Placebo-Controlled, Crossover Trial

**DOI:** 10.3390/nu11030640

**Published:** 2019-03-16

**Authors:** Rebeca Quirós-Fernández, Bricia López-Plaza, Laura M. Bermejo, Samara Palma-Milla, Carmen Gómez-Candela

**Affiliations:** 1 Nutrition Research Group, Hospital La Paz Institute for Health Research (IdiPAZ), 28046 Madrid, Spain; rquirosfernandez@gmail.com (R.Q.-F.); laura.bermejol@salud.madrid.org (L.M.B.); 2 Nutrition Department, La Paz University Hospital, Hospital La Paz Institute for Health Research (IdiPAZ), Autonomous University of Madrid, 28046 Madrid, Spain; samara.palma@salud.madrid.org (S.P.-M.); cgcandela@salud.madrid.org (C.G.-C.)

**Keywords:** atherosclerosis, hydroxytyrosol, punicalagin, endothelial dysfunction, oxidative stress, prehypertension, hypertension

## Abstract

Hydroxytyrosol (HT) and Punicalagin (PC) exert cardioprotective and anti-atherosclerotic effects. This study evaluates the effect of oral supplementation with HT and PC (SAx) on early atherosclerosis markers in middle-aged, seemingly healthy adults. A randomized, double-blinded, placebo-controlled, crossover trial was performed for 20 weeks. There were two treatment sequences (Placebo/SAx, n = 41; SAx/Placebo, n = 43) for which the intervention periods (Placebo and SAx) were 8 weeks long, followed by a 4-week wash out period. The supplement was composed of 9.9 mg of HT and 195 mg of PC, and the placebo was composed of maltodextrin. SAx increased endothelial function (Flow-mediated dilatation [FMD]: 2.36%; *p* < 0.001) in the endothelial dysfunction subgroup compared to the placebo (2.36 ± 3.9 vs. 0.76 ± 3.5%, *p* < 0.05). SAx also reduced oxLDL by −28.74 ng/mL (*p* < 0.05) in subjects with higher levels of oxLDL, which was an improvement compared with the placebo (−28.74 ± 40.2 vs. 25.64 ± 93.8 ng/mL, *p* < 0.001). The prehypertension and hypertension subgroups exhibited decreased systolic (−15.75 ± 9.9 mmHg; *p* < 0.001) and diastolic (−6.36 ± 8.7 mmHg; *p* < 0.001) blood pressure after SAx consumption. Moreover, the systolic prehypertension and hypertension subgroups presented significant differences in systolic blood pressure compared to the placebo (−15.75 ± 9.9 vs. −2.67 ± 12.0 mmHg, *p* < 0.05). In conclusion, the supplement exerted anti-atherosclerotic effects by improving endothelial function, blood pressure, and levels of circulating oxLDL, especially for persons in whom these parameters were altered.

## 1. Introduction

Cardiovascular disease (CVD) is the leading cause of death worldwide. The annual mortality from CVD is higher than from any other cause [1,2]. In the last 40 years, a rapid increase in the prevalence of cardiovascular diseases has been observed. In 2030, it is estimated that approximately 23.3 million people will die from CVD, which is likely to remain the world’s foremost cause of death [3]. Most cases of CVD, however, are preventable through the reduction of behavioural risks (tobacco use, unhealthy diet, physical inactivity or harmful use of alcohol) [1,4].

Atherosclerosis, which is also known as atherosclerotic vascular disease (ASVD), is one of the most important causes of CVD. The development of atherosclerosis involves a number of pathophysiological mechanisms [5,6], including endothelial dysfunction (ED) [7]. ED, which is greatly underdiagnosed in the general population, plays a pivotal role in the early stages of ASVD, and is later associated with plaque progression and the occurrence of cardiovascular (CV) events [8]. Flow-mediated dilatation (FMD) is an accepted technique to quantify endothelial function and has been shown to have prognostic value to future CV events in symptomatic [9,10] and apparently healthy or asymptomatic subjects [11,12,13].

With regard to blood pressure, it is well known that prehypertension often leads to hypertension [14,15] and an increased risk of CVD and future CV events [16]. Furthermore, the relationship between blood pressure and the risk of CV events is continuous, consistent, and independent of other cardiovascular risk factors [17]. Therefore, the prevention and treatment of hypertension is key for reducing the burden of CV morbi-mortality [18,19].

The role of oxidative stress (OS) in the pathophysiology of CVD is well established [6,20,21]. OS is one of the primary mechanisms involved in the development of ASVD. The circulating level of oxidised low-density lipoproteins (oxLDL) is one of the most important markers in the atherogenic process [22]. These lipoproteins contribute significantly to the development of ASVD, and they not only promote the formation of atherosclerotic plaque [23], but also participate in its progression [24,25,26] and destabilization [27,28,29,30]. This action is carried out through several mechanisms, including the promotion of ED [31,32,33,34]. Consequently, elevated levels of circulating oxLDL are associated with all stages of atherosclerosis, from the peripheral early atherogenesis stage, through coronary artery disease, until acute coronary syndromes and ischaemic stroke [22]. Therefore, the level of circulating oxLDL is a predictor of future CV events in apparently healthy or asymptomatic subjects [35] and in symptomatic subjects [27,29].

Polyphenols are gaining increasing acceptance as therapeutic agents for use in diverse diseases, including CVD [36] and its risk factors [37,38,39,40,41,42]. Several studies have reported an inverse correlation between the consumption of polyphenols and the risk of CV events [36] and overall mortality [40]. Among these compounds, hydroxytyrosol (HT, from olives) [43,44,45] and punicalagin (PC, from pomegranates) [46,47] have been attributed as having cardioprotective properties. The intake of adequate amounts of these bioactive compounds may therefore help reduce the morbidity-mortality associated with CVD.

The aim of the present work was to determine the effect of supplementation with HT and PC on endothelial dysfunction and other early atherosclerosis markers in apparently healthy middle-aged subjects.

## 2. Materials and Methods

The present study was registered at http://clinicaltrials.gov under the number NCT02042742.

### 2.1. Study Subjects

One hundred and five subjects aged 45–65 years were recruited for the present study by the Nutrition Department of La Paz University Hospital (HULP), Madrid (Spain). The inclusion criteria to be eligible for the study were as follows: aged between 45 and 65 years, having a suitable understanding of the clinical trial level, agreeing to voluntarily participate in the study, and signing the informed consent. The exclusion criteria were as follows: a BMI of ≥30 kg/m^2^; subjects receiving drug treatment for CV risk (dyslipidaemia, hypertension, diabetes mellitus, and others); those who had been diagnosed with metabolic syndrome, who had a mental illness or low cognitive ability; and those who suffered severe liver or kidney disease, or cancer. Subjects were also excluded if they had a family background of premature vascular disease, or if they were taking supplements (antioxidants, ω-3, vitamins, minerals, or probiotics). Women still experiencing menstrual cycles and subjects who planned to stop smoking or lose weight during the study were also excluded, as were subjects who had problems with complying with the general dietary recommendations, those who undertook intensive physical activity, those who consumed >30 g/day alcohol, and those who had allergies to olive and pomegranate by-products.

All subjects gave their informed consent to take part in the study, which was approved by the Scientific Research and Ethics Committee of the HULP (Code 3799) in accordance with the ethical standards of the Declaration of Helsinki [48].

### 2.2. Study Design

The study took the form of a randomized, controlled, double-blind, crossover clinical trial lasting 20 weeks. Subjects (*n* = 84) were randomly assigned (maintaining the gender ratio of the sample) to one of two treatment sequences involving an oral supplementation (9.9 mg of HT plus 195 mg of PC, and 995.1 mg of maltodextrin per day) or a placebo (1.200 mg of maltodextrin per day) for 8 weeks, followed by a 4-week wash-out interval and then a cross-over. During the 8-week intervention period, subjects consumed 3 capsules/day (containing either the oral supplement SAx or the placebo) with their meals. Neither the researchers nor the subjects knew which treatment sequence the subjects had been assigned to; the researchers were unblinded only at the end of the study.

### 2.3. Supplement and Placebo Capsules

The doses administered in the present work were in agreement with those reported in the literature [44,49,50,51]. Each supplement capsule (SAx) contained 3.3 mg of HT from a standardized olive fruit extract (Mediteanox^®^, Euromed S.A., Barcelona, Spain), 65 mg of PC from a standardized pomegranate fruit extract (Pomanox^®^ P30, Euromed S.A., Barcelona, Spain), and 331.7 mg of maltodextrin. Each Placebo capsule contained 400 mg of maltodextrin. Probelte Pharma S.A. (Murcia, Spain) prepared the supplement capsules (with specified concentrations of HT and PC) and the placebo capsules specifically for this study. Both types of capsules were packaged in blister packs of 15, and the blister packs were labelled as either L1 or L2 to maintain blinded conditions. Subjects received all the capsules needed to complete the 8-week intervention period (SAx or placebo) during repeat appointments at the HULP.

### 2.4. Methods

#### 2.4.1. Diet

All subjects were told to maintain their normal dietary habits and not to increase their consumption of foods rich in antioxidants. The diet of each subject was recorded during the week prior to the beginning and end of each intervention period. All food and beverages consumed inside and outside the home were recorded over three consecutive days (including one day of the weekend) [52]. Subjects were instructed to record the weight of the food consumed or, if this was not possible, to record household measurements (spoonfuls, cups, etc.). At each visit, all records were thoroughly reviewed by a nutritionist in the presence of the subject to ensure that the information collected was complete. The energy and nutritional content of the foods and beverages consumed were then calculated using DIAL software (Alce Ingeniería, Madrid, Spain).

#### 2.4.2. Anthropometric Variables

Anthropometric measurements were taken at the beginning and end of each intervention period using standard techniques, adhering to international norms set out by the WHO [53]. All measurements were made by trained personnel in the morning with the subject barefoot and wearing only underwear. Body composition was determined using a specialized bioelectrical impedance analyser, the EFG ElectroFluidGraph analyser (Akern s.r.l., Florence, Italy). Height was determined using a height meter with an accuracy of 1 mm (range, 80–200 cm). BMI was calculated using the following formula: [weight (kg)/height (m)^2^].

#### 2.4.3. Health Variables

Information was collected on medical conditions and the consumption of medications. Blood pressure and heart rate were measured on the right arm using a Spot Vital Signs 420 automatic monitor (Welch Allyn, Madrid, Spain) (accuracy ±5 mmHg). Three measurements were taken at 5-min intervals, and the means were calculated. Populations were classified according to whether the subjects exhibited prehypertension or high blood pressure that was non-diagnosed and was not being treated pharmacologically.

#### 2.4.4. Determination of Vascular Function Variables

Flow-mediated dilatation (FMD) in the brachial artery was used as an indicator of endothelial function. FMD has been shown to be an independent predictor of CV events [9,10], even in apparently healthy subjects [11,12,13]. Measurements were performed based on recommendations described in published guidelines [54] using a Doppler ultrasound device with a 10-MHz linear probe (Biosound MyLab 25 Portable Ultrasound System, Esaote, Genoa, Italy). Briefly, the subject lay in the supine position in a room at 20–22 °C. A sphygmomanometer cuff was positioned just below the axilla, and the Doppler probe was placed 3 cm above the right elbow (with the arm extended) at an angle of 60 °. After a 10-min resting period, basal capillary flow was measured (t0). Thereafter, distal ischaemia was induced for 5 min by inflating the cuff to a pressure of 250 mmHg. The cuff was then deflated and, after 70 s, the flow was recorded (td). All images were captured by the same qualified operator. All variables were measured in triplicate, and their means were calculated. The area under the curve (AUC) for t0 and td was then determined. The FMD was calculated using the following equation: FMD = (AUCtd − AUCt0) × 100/AUCt0, and the subjects were classified according to the presence or absence of ED (ED = FMD < 10%) [55].

#### 2.4.5. Biochemical Data

At the beginning and end of each intervention period, blood samples were collected early in the morning at the La Paz University Hospital Extraction Unit. Samples were kept at 4–6 °C until analysis, which was always performed within 48 h. The concentration of plasma-soluble vascular cell adhesion molecule-1 (sVCAM-1) was determined using a Luminex^®^ 200^TM^ multianalyte profiling system and commercially available immunoassay panels in the HCVD2MAG-67K Milliplex Map Kit (EMP Millipore Corp., Boston, MA, USA). Plasma Ox-LDL levels were measured using ELISA with human apoB-100 modified by Malondialdehyde (MDA-LDL) (Immundiagnostik AG, Bensheim, Germany).

Total plasma lipid peroxides were measured using the thiobarbituric acid reactive substances (TBARS) method [56]. Plasma antioxidant capacity was analysed using a ferric reducing antioxidant power (FRAP) assay [57]. Quantitative measurements of Paraoxonase-1 (PON-1) were made using a Human Serum PON-1 ELISA Kit (Aviscera Bioscience, Santa Clara, CA, USA). Plasma 8-isoprostanes (8-iso-PGF_2α_) was measured using an OxiSelect^TM^ 8-iso-PGF_2α_ ELISA kit (Cell Biolabs, San Diego, CA, USA). Nitrate/Nitrite (NOx) was analysed in plasma using a commercially available colorimetric assay kit (Cayman Chemical Company, Ann Arbor, MI, USA) according to the Griess method [58].

#### 2.4.6. Compliance and Adverse Events

Subjects received the exact number of capsules (in blister packaging) required for each intervention period during a pre-period appointment, and they were asked to return all empty and non-empty blister packages. Compliance was measured at the middle of each intervention period during an interview and at the end of each intervention period by comparing the number of capsules provided and the number returned. A subject was considered compliant when he/she consumed ≥90% of the capsules provided. Adverse events were recorded on the final visit of each intervention period. An adverse event was defined as any unfavourable, unintended effect. All such events were recorded along with the symptoms involved (nausea, vomiting, diarrhoea, and constipation).

### 2.5. Statistical Analysis

A sample size of 38 patients was calculated as the sample size that was necessary to provide 90% power (at α = 0.05) to determine an absolute difference of 2% in FMD [59] (potential 20% dropout included). Quantitative data are presented as the means ± standard deviations (SD). Qualitative data are presented as counts and percentages. The Kolmogorov–Smirnov test was used to determine whether the data were normally distributed. Levene’s test was used to assess the equality of variance. The denominator degree of freedom was approximated using the Satterthwaite formula. The type class variables considered in the primary model included the treatment, period, treatment × period interaction, risk, and the treatment × risk interaction; these variables were added as fixed effects. The type class variables considered in the second model included the start–end, treatment, start–end × treatment interaction, period, treatment × period interaction, start–end × treatment × period interaction, the risk, treatment × risk interaction, and the start–end × treatment × risk interaction; these variables were added as fixed effects. For both models, the subject was considered in sequence as a random effect and a “variance components” covariance matrix. The type III tests of fixed effects table lists the hypothesis tests for the significance of each of the fixed effects specified in both models; in particular, the carryover effect was assessed in the primary model by the treatment × period interaction effect. If the carryover effect was significant for a given variable, then only the first period was considered for the analysis. Least squares means of the primary model were computed and compared for the treatment and the treatment × risk interaction variables. Least squares means of the second model were computed and compared for the treatment × start–end interaction and the start–end × treatment × risk interaction. Multiple comparisons were adjusted using the Bonferroni method. Two-sided tests were used, and a *p*-value < 0.05 was considered statistically significant. Statistical analyses were performed using the linear mixed model in the SAS statistical software package, version 9.3 (SAS Institute Inc., Cary, NC, USA).

## 3. Results

### 3.1. Recruitment and Study Population

**Figure 1:**
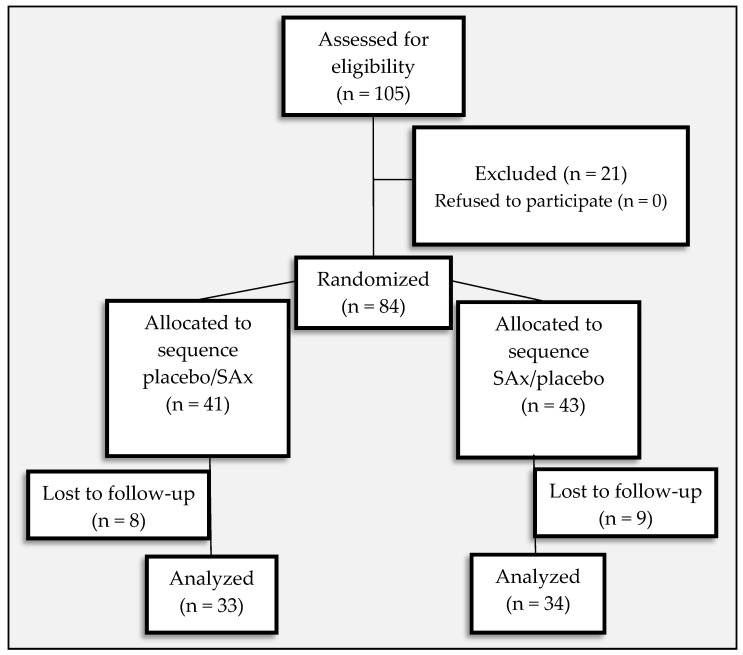
Flow chart describing the present trial.

The study was performed between February and June 2013. Eighty-four apparently healthy subjects (17 men [20.2%], 67 women [79.8%]) were eligible for inclusion. Seventeen subjects were lost to follow-up (8 in the placebo/SAx sequence and 9 in the SAx/placebo sequence) due to personal causes (*n* = 15) and failure to follow treatment instructions (*n* = 2). Thus, 67 subjects (14 men [20.9%], 53 women [79.1%]) completed the 20-week study, and only their results were included in the subsequent analyses (Figure 1).

### 3.2. Baseline Characteristics

The mean age of the population was 53.0 ± 4.5 years old. The mean BMI was 24.6 ± 3.1 kg/m^2^. At the start of the study, no significant differences existed between subjects assigned to the supplementation (SAx) and placebo sequences in terms of their anthropometric, vascular function, oxidative status, and other variables (gender, age, and smoking) (Table 1).

### 3.3. Dietetic and Anthropometric Variables

No significant differences were detected in any dietetic or anthropometric variables between the start and end of the intervention periods, nor between the periods in terms of changes in these variables (Table 2).

### 3.4. Vascular Function Variables

Table 3 shows the values of the vascular function variables examined. A significant reduction in systolic blood pressure (SBP) was observed at the end of the SAx period (SAx period—start 111.3 ± 12.9, end 101.9 ± 12.0 mmHg, *p* < 0.001). The reduction in SBP observed after the supplementation treatment was significantly greater than that recorded for the placebo treatment (SAx −9.42 ± 10.3 vs. placebo −2.79 ± 11.2 mmHg, *p* < 0.05). After the SAx treatment, subjects with systolic prehypertension or hypertension exhibited significantly decreased SBP (SAx period—start 123.9 ± 4.2, end 108.2 ± 10.8 mmHg, *p* < 0.001); no such significant reduction was observed at the end of the placebo period (from 129.8 ± 2.8 to 127.2 ± 10.4 mmHg). In addition, in these subjects with systolic prehypertension or hypertension, the reduction in SBP that was observed following the SAx treatment was significantly greater than that recorded for the placebo treatment (SAx −15.75 ± 9.9 vs. placebo −2.67 ± 12.0 mmHg, *p* < 0.05).

A significant reduction in diastolic blood pressure (DBP) was also observed at the end of the SAx period (from 74.34 ± 10.1 to 71.60 ± 10.4 mmHg, *p* < 0.001); no such significant reduction was observed at the end of the placebo period (from 72.68 ± 10.1 to 71.42 ± 9.7 mmHg). These improvements were observed as well in the subgroup of diastolic prehypertension and hypertension after SAx treatment (from 84.4 ± 6.5 to 78.04 ± 10.8 mmHg, *p* < 0.001); no such improvement was observed after the placebo period (from 83.0 ± 5.6 to 79.9 ± 8.8 mmHg, NS). Subjects with normal blood pressure remained stable.

**Figure 2:**
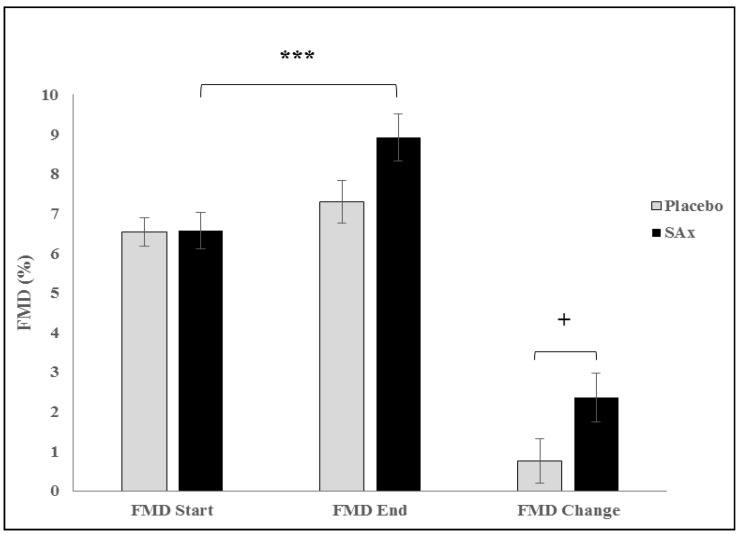
Endothelial Function (FMD), which is a predictor of future cardiovascular events in symptomatic and asymptomatic subjects, significantly increased following SAx (Oral Supplementation with HT and PC) treatment (black color) in apparently healthy subjects (*** *p* < 0.001), and this improvement was significantly different compared to that observed following the placebo (grey) (+*p* < 0.05). Forty-five subjects had endothelial dysfunction. Data represent the adjusted means (±SD) from multivariate models.

Significant differences were also observed in the FMD between the start and the end of the SAx period (from 8.04 ± 4.0 to 9.46 ± 4.0%, *p* < 0.05); no such improvement was observed for the placebo period (from 8.08 ± 3.3 to 8.62 ± 4.0%, NS). In subjects with ED, a significant increase in FMD was observed after the SAx period (from 6.57 ± 2.9 to 8.93 ± 3.8%, *p* < 0.001) but not after the placebo period (from 6.54 ± 2.3 to 7.30 ± 3.4%, NS). The increase in FMD that was observed following the SAx treatment was significantly greater than that recorded for the placebo treatment (SAx 2.36 ± 3.9 vs. placebo 0.76 ± 3.5 %, *p* < 0.05) (Figure 2). No significant changes in FMD were observed in subjects without ED in either intervention period (SAx from 10.86 ± 4.5 to 10.70 ± 4.2%; placebo from 11.16 ± 2.9 to 11.26 ± 3.7%). No differences were recorded in heart rate (HR) or soluble sVCAM-1 in any comparison.

### 3.5. Oxidative Status Variables

Table 4 shows the values of the oxidative status variables recorded. Circulating oxLDL levels were reduced significantly after the SAx period (from 108.9 ± 126.2 to 97.44 ± 121.7 ng/mL, *p* < 0.05); however, no significant reduction was recorded after the placebo period (from 98.86 ± 128.1 to 105.9 ± 139.9 ng/mL). The reduction in oxLDL observed after the SAx treatment was significantly greater than that recorded for the placebo treatment (SAx −11.46 ± 28.1 vs. placebo 7.05 ± 55.6 ng/mL, *p* < 0.05). In subjects with higher levels of oxLDL, these levels were reduced significantly after the SAx treatment (from 258.2 ± 138.0 to 229.5 ± 149.5 ng/mL, *p* < 0.05); no significant reductions were observed after the placebo period (from 235.4 ± 162.5 to 261.0 ± 170.8 ng/mL). In these subjects, the reduction in oxLDL that was observed following the SAx period was significantly greater than that recorded for the placebo treatment (−28.74 ± 40.2 vs. 25.64 ± 93.8 ng/mL, *p* < 0.001). No significant differences were observed for the remaining variables.

### 3.6. Compliance and Adverse Events

All subjects ingested >90% of the capsules provided. No significant differences were observed in the number of capsules consumed between the different intervention periods or treatment sequences. No adverse events resulting from the intake of either type of treatment capsule were reported.

## 4. Discussion

This is the first clinical trial to study the effect of the regular intake of a supplement rich in HT and PC on early atherosclerosis markers in middle-age subjects. The consumption of three capsules per day containing HT and PC (9.9 mg of HT and 195 mg of PC) for 8 weeks significantly improved endothelial function and blood pressure and reduced LDL oxidation in middle-aged subjects. These improvements were observed primarily in subjects who were at risk of developing cardiovascular disease. No adverse effects were observed.

Several studies have shown that HT [43,44,45,60,61] and PC [46,47] possess cardioprotective properties that may reduce the risk of developing CVD. However, most of these studies were performed in vitro or with experimental animals [24,25,26,27,28]; some studies have investigated the effects of these compounds in humans [60,62], and no studies have examined the combined effect of HT and PC.

The present results reveal a significant improvement in FMD among subjects with ED. One of the major objectives of a CVD detection programme must be to identify those apparently healthy subjects who have asymptomatic arterial disease; such identification may reduce the risk, delay the onset, and perhaps even induce the regression of atherosclerotic disease. The importance of new imaging methods for the detection of subjects at a high risk for suffering CV events has been recognized [63]. FMD is a non-invasive imaging technique that is accepted for quantifying endothelial function; in addition, FMD is the most widely used technique for testing endothelial function. FMD has been shown to have prognostic value for predicting future CV events in symptomatic subjects [9,10] and apparently healthy or asymptomatic subjects [11,12,13]. In fact, Kuvin et al. [55] observed that FMD measurements of the brachial artery were highly predictive of CVD, with an increase in the odds ratio of 1.32 for each percentage decrease in FMD below 10% [55]. The FMD also has a significant potential as a screening tool for CVD in individuals with an apparent low CV risk assessed by traditional risk factors [55]. The improvement in endothelial function from HT, PC, or foods rich in these compounds has been observed in many studies [41,47,62,64]. In addition, some authors suggest a possible inverse relationship between FMD and oxLDL levels [31,41]. In the present study, simultaneous improvements in FMD and oxLDL were observed.

It therefore appears that subjects with ED could benefit from supplementation with HT and PC, even if they are not diagnosed. In this regard, there remains a high prevalence of undiagnosed ED. Indeed, in this study of apparently healthy subjects, the percentage of subjects who were eventually diagnosed with ED reached 69.2%. In subjects with ED, these benefits could potentially help to reduce their risk of CVD and the future burden they may pose to the health system. In the present work, SBP and DBP significantly improved in the total population after intake of the supplement for 8 weeks. However, this effect was observed primarily in subjects suffering from prehypertension or hypertension. This finding is important because, in addition to hypertension, prehypertension also increases the risk of CVD and CV events [16]. Hypertension is usually inadequately controlled [65], and antihypertensive drugs can produce adverse effects that often lead to patient abandonment [66]. Therefore, the prevention and treatment of hypertension is key for reducing the burden of CV morbi-mortality [18,19]. In this context, various studies in which HT or PC were provided via functional foods or foods rich in these compounds also reported significant improvements in blood pressure [39,67]. Even the European Medicines Agency (EMA) recognized the hypotensive activity of olive leaf; however, the EMA mentioned that there are not sufficient data from well-designed clinical trials to support this indication [68]. Therefore, the present study contributes additional evidence suggesting that the hypotensive action after the consumption of foods, functional foods, or supplements containing HT or PC (or both) may help regulate blood pressure.

On the other hand, numerous studies have reported a reduction in oxLDL levels after the intake of foods rich in HT or PC [40,44,67,69,70,71]. In a crossover clinical trial in healthy subjects, it was reported that the consumption of 25 mL/day virgin olive oil (~3.75 mg/day phenolic compounds) for 3 weeks significantly reduced plasma oxLDL levels [69]. In the EUROLIVE study, a crossover clinical trial in healthy subjects reported that the consumption of 25 mL/day high-phenolic-content virgin olive oil (366 mg/L phenolic content) for 3 weeks induced a greater reduction in oxLDL levels compared with olive oils with a lower phenolic content [40]. In this regard, the EFSA recognized a cause and effect relationship between the daily consumption of 5 mg of HT and its derivatives from olive oil and the protection of c-LDL particles from oxidative damage [44]. The results of the present work are in line with these studies regarding HT as a protection strategy against c-LDL oxidative damage [44]. One mechanism through which HT may reduce oxLDL levels is by modulating the expression of genes involved in atherogenesis [45,72]. Other authors have reported that the long-term consumption of 50 mL/day pomegranate juice (~85 mg of PC) significantly reduces oxLDL levels in patients with carotid artery stenosis [67]. PC may provide this benefit through a direct antioxidant effect on c-LDL, by increasing serum PON-1 levels, and/or by reducing the capacity of macrophages to oxidize c-LDL [67,70,71]. However, in the present work, although supplementation with HT and PC reduced the formation of oxLDL, no change was observed in serum PON-1 levels or, indeed, in any other oxidative status variables (TBARS, FRAP, 8-iso-PGF_2α_, and NOx). This discrepancy may be explained by the fact that previous studies have involved subjects with CVD-related pathologies, whereas the subjects in the present study were apparently healthy. Similarly, PON-1 activity has been observed to be decreased in subjects with diabetes [73] but not in those with pre-diabetes or a new diagnosis of diabetes [74].

A high level of circulating oxLDL is a marker of ED [31] and a predictor of future CVD events in apparently healthy or asymptomatic subjects [35]. Therefore, the regular intake of supplements containing both HT and PC, which reduce the formation of oxLDL in subjects with elevated oxLDL levels, may help to reduce the CV risk in these subjects.

One possible limitation of this study is the sample size.

## 5. Conclusions

In summary, the consumption of a supplement containing HT and PC (9.9 mg of HT and 195 mg of PC per day) for 8 weeks could help reducing c-LDL oxidation and improved SBP, DBP, and FMD in middle-aged subjects. These improvements in FMD, blood pressure, and circulating oxLDL levels were most pronounced in subjects with alterations in these atherosclerotic markers. Therefore, the regular intake of a supplement containing HT and PC may reduce the risk of CV in these subjects. Further clinical trials are warranted to confirm the beneficial effect of these polyphenols in humans.

## Figures and Tables

**Table 1:** Baseline characteristics of the subjects.

		Placebo/SAx (*n* = 33)	SAx/Placebo (*n* = 34)
Gender	(Female %, *n*)	78.79 (26)	79.41 (27)
Age	(years)	53.21 ± 4.2	52.79 ± 4.8
Smoking	(Smokers %, *n*)	18.18 (6)	26.47 (9)
Weight	(kg)	66.26 ± 11.8	64.08 ± 10.9
BMI	(kg/m^2^)	24.64 ± 2.9	24.56 ± 3.2
Waist circumference	(cm)	80.51 ± 9.2	82.58 ± 9.8
FM	(%)	29.18 ± 6.7	28.76 ± 6.4
FFM	(%)	70.82 ± 6.7	71.24 ± 6.4
MM	(%)	48.03 ± 7.7	47.87 ± 5.5
SBP	(mmHg)	110.3 ± 13.1	110.9 ± 12.9
DBP	(mmHg)	74.06 ± 10.8	73.75 ± 9.5
HR	(bpm)	67.36 ± 8.9	70.41 ± 7.5
FMD	(%)	8.464 ± 4.0	7.962 ± 3.8
sVCAM-1	(pg/mL)	679.9 ± 191.8	661.5 ± 183.1
oxLDL	(ng/mL)	118.2 ± 144.4	103.4 ± 113.9
TBARS	(µmol MDA eq/mL)	0.622 ± 0.8	0.572 ± 0.7
FRAP	(µmol Trolox eq/mL)	0.410 ± 0.1	0.425 ± 0.1
8-iso-PGF_2α_	(ng/mL)	58.55 ± 12.6	56.95 ± 10.3
PON-1	(ng/mL)	5.777 ± 6.5	4.383 ± 3.9
NOx	(µM)	23.28 ± 9.0	27.16 ± 19.9

Data are expressed as the means ± SDs. SAx: Oral Supplementation with HT and PC; BMI: Body Mass Index; FM: Fat mass; FFM: Fat-Free Mass; MM: Muscle Mass; SBP: Systolic Blood Pressure; DBP: Diastolic Blood Pressure; HR: Heart Rate; FMD: Flow Mediated Dilatation; sVCAM-1: soluble Vascular Cell Adhesion Molecule-1; oxLDL: Oxidised Low-Density Lipoprotein; TBARS: Thiobarbituric Acid Reactive Substances; FRAP: Ferric Reducing Antioxidant Power; 8-iso-PGF_2α_: 8-iso-prostaglandinF_2α_; PON-1: Paraoxonase-1; NOx: Nitrates & Nitrites. There are not significant differences between the start and end of the intervention periods or in the change recorded between the intervention periods.

**Table 2:** Dietetic or anthropometric variables at the start and end of the supplementation and placebo periods.

			SAx	Placebo
			(*n* = 67)	(*n* = 67)
Energy	(kcal/day)	Start	1923 ± 513.6	1864 ± 471.9
		End	1891 ± 549.4	1881 ± 569.5
		Change	−31.88 ± 463.2	17.07 ± 355.5
Carbohydrates	(%)	Start	38.06 ± 6.5	38.46 ± 8.9
		End	37.62 ± 6.3	39.06 ± 6.5
		Change	−0.439 ± 5.7	0.598 ± 8.1
Proteins	(%)	Start	17.24 ± 3.7	17.44 ± 2.9
		End	17.43 ± 3.6	17.60 ± 3.1
		Change	0.193 ± 4.4	0.164 ± 3.5
Lipids	(%)	Start	41.42 ± 6.3	40.48 ± 8.8
		End	41.36 ± 5.7	40.19 ± 5.9
		Change	−0.067 ± 5.3	−0.287 ± 8.0
Weight	(kg)	Start	65.10 ± 11.3	65.10 ± 11.2
		End	64.93 ± 11.2	64.85 ± 11.3
		Change	−0.173 ± 1.3	−0.249 ± 1.0
BMI	(kg/m^2^)	Start	24.58 ± 3.0	24.63 ± 3.0
		End	24.51 ± 3.0	24.48 ± 3.0
		Change	−0.068 ± 0.5	−0.151 ± 0.6
Waist	(cm)	Start	81.85 ± 9.0	81.24 ± 9.8
circumference		End	81.82 ± 9.6	81.25 ± 9.6
		Change	−0.034 ± 2.9	0.008 ± 3.7
FM	(%)	Start	29.16 ± 6.6	28.90 ± 6.5
		End	29.56 ± 6.8	29.79 ± 7.2
		Change	0.400 ± 2.8	0.891 ± 3.7
FFM	(%)	Start	70.84 ± 6.6	71.10 ± 6.5
		End	70.44 ± 6.8	70.21 ± 7.2
		Change	−0.400 ± 2.8	−0.891 ± 3.7
MM	(%)	Start	47.63 ± 6.2	47.52 ± 6.5
		End	46.67 ± 5.7	46.44 ± 5.8
		Change	−0.970 ± 5.1	−1.082 ± 5.8

Data are expressed as the means ± SDs. SAx: Oral Supplementation with HT and PC; BMI: Body Mass Index; FM: Fat mass; FFM: Fat-Free Mass; MM: Muscle Mass. There are not significant differences between the start and end of the intervention periods or in the change recorded between the intervention periods.

**Table 3:** Vascular function variables at the start and end of the supplementation and placebo periods.

			SAx	Placebo
			(*n* = 67)	(*n* = 67)
SBP	(mmHg)	Start	111.3 ± 12.9	110.2 ± 13.3
		End	101.9 ± 12.0 ***	107.4 ± 14.8
		Change	−9.419 ± 10.3 #	−2.792 ± 11.2
DBP	(mmHg)	Start	74.34 ± 10.1	72.68 ± 10.1
		End	71.60 ± 10.4 ***	71.42 ± 9.7
		Change	−2.742 ± 8.4	−1.258 ± 8.1
HR	(bpm)	Start	68.63 ± 10.7	69.13 ± 9.4
		End	67.67 ± 9.8	67.88 ± 8.4
		Change	−0.962 ± 8.3	−1.250 ± 9.1
FMD	(%)	Start	8.036 ± 4.0	8.077 ± 3.3
		End	9.462 ± 4.0 *	8.619 ± 4.0
		Change	1.427 ± 3.7	0.542 ± 3.4
sVCAM-1	(pg/mL)	Start	650.8 ± 192.9	635.3 ± 174.9
		End	625.0 ± 163.9	588.7 ± 136.7
		Change	−25.84 ± 175.1	−46.66 ± 126.6

Data are expressed as the means ± SDs. SAx: Oral Supplementation with HT and PC; SBP: Systolic Blood Pressure; DBP: Diastolic Blood Pressure; HR: Heart Rate; FMD: Flow Mediated Dilatation; sVCAM-1: soluble Vascular Cell Adhesion Molecule-1. Significant differences between the start and end of the intervention periods (* *p* < 0.05, *** *p* < 0.001). Significant differences in the change recorded between the intervention periods (# *p* < 0.05).

**Table 4:** Oxidative status at the start and end of the supplementation and placebo periods.

			SAx	Placebo
			(*n* = 67)	(*n* = 67)
oxLDL	(ng/mL)	Start	108.9 ± 126.2	98.86 ± 128.1
		End	97.44 ± 121.7 *	105.9 ± 139.9
		Change	−11.46 ± 28.1 #	7.05 ± 55.6
TBARS	(µmol MDA eq/mL)	Start	0.696 ± 0.9	0.673 ± 0.9
		End	0.635 ± 0.9	0.574 ± 0.8
		Change	−0.061 ± 1.2	−0.099 ± 1.1
FRAP	(µmol Trolox eq/mL)	Start	0.421 ± 0.1	0.422 ± 0.1
		End	0.425 ± 0.1	0.433 ± 0.1
		Change	0.005 ± 0.1	0.011 ± 0.0
8-iso-PGF_2α_	(ng/mL)	Start	52.54 ± 11.7	54.06 ± 13.6
		End	50.68 ± 14.0	50.55 ± 11.7
		Change	−1.858 ± 13.7	−3.507 ± 14.9
PON-1	(ng/mL)	Start	4.187 ± 4.4	4.130 ± 5.5
		End	3.567 ± 5.1	4.514 ± 7.0
		Change	−0.619 ± 6.5	0.384 ± 7.7
NOx	(µM)	Start	25.34 ± 16.2	23.57 ± 13.5
		End	26.39 ± 16.9	22.52 ± 9.7
		Change	1.053 ± 14.4	−1.055 ± 13.1

Data are expressed as the means ± SDs. SAx: Oral Supplementation with HT and PC; OxLDL: Oxidised Low-Density Lipoprotein; TBARS: Thiobarbituric Acid Reactive Substances; FRAP: Ferric Reducing Antioxidant Power; 8-iso-PGF_2α_: 8-iso-prostaglandinF_2α_; PON-1: Paraoxonase-1; NOx: Nitrates & Nitrites. Significant difference between the start and end of the intervention periods (* *p* < 0.05). Significant differences in the change recorded between the intervention periods (# *p* < 0.05).

## Abbreviations

8-iso-PGF2α	8-isoprostanes
ASVD	Atherosclerotic Vascular Disease
AUC	Area Under the Curve
CV	Cardiovascular
CVD	Cardiovascular Disease
DBP	Diastolic Blood Pressure
ED	Endothelial Dysfunction
ELISA	Enzyme-Linked-Immunosorbent-Assay
FMD	Flow-Mediated Dilatation
FRAP	Ferric Reducing Antioxidant Power
HR	Heart Rate
HT	Hydroxytyrosol
HULP	University Hospital La Paz
OS	Oxidative Stress
oxLDL	oxidized Low Density Lipoprotein Cholesterol
PC	Punicalagin
PON-1	Paraoxonase-1
SBP	Systolic Blood Pressure
sVCAM-1	soluble Vascular Cell Adhesion Molecule-1
TBARS	Thiobarbituric Acid Reactive Substances
